# Coronary tortuosity relation with carotid intima-media thickness, coronary artery disease risk factors, and diastolic dysfunction: is it a marker of early atherosclerosis?

**DOI:** 10.1186/s43044-021-00157-6

**Published:** 2021-03-31

**Authors:** Ahmed Elamragy, Samuel Yakoub, Mohamed AbdelGhany, Waleed Ammar

**Affiliations:** 1 Department of Cardiology, Kasr Al Aini Hospital, Faculty of Medicine, Cairo University, Cairo, 11562 Egypt; 2 Department of Cardiology, 6th Of October Health Insurance Hospital, Giza, 12573 Egypt

**Keywords:** Coronary, Tortuosity, Carotid, Intima-media thickness, atherosclerosis

## Abstract

**Background:**

Coronary tortuosity (C-Tor) is a common finding in coronary angiography (CAG). There are conflicting data about its link to atherosclerosis: one study found a negative relationship with coronary artery disease (CAD), although it had been linked to age and hypertension (HTN), which are CAD risk factors. Carotid intima-media thickness (C-IMT) is a measure of early atherosclerosis and a surrogate for CAD, diastolic dysfunction is also associated with CAD risk factors. In this retrospective case-control study, we investigated the relationship between C-Tor, C-IMT, diastolic dysfunction, and the other risk factors in patients undergoing CAG in a tertiary hospital between July 2017 and June 2018, after excluding patients with significant CAD. C-Tor was defined as the presence of ≥ 3 bends (≥ 45°) along the trunk of at least one main coronary artery in CAG.

**Results:**

After excluding 663 patients due to exclusion criteria, 30 patients with C-Tor were compared with age and gender-matched controls. HTN was significantly more common in the C-Tor group (86.7% vs. 30%, *p* < 0.002); other clinical characteristics were similar. The C-IMT was abnormal in the C-Tor group only (*p*: 0.007). The diastolic dysfunction parameters differed between the two groups: the E/A ratio was < 1 in the C-Tor group and > 1 in the normal group (*p*: < 0.001); the E velocity and deceleration time were significantly lower in the C-Tor group (*p*: 0.001 and < 0.001 respectively); the E/E′ ratio, A, and A′ velocities were significantly higher (*p*: 0.005, < 0.001, < 0.001 respectively); while the S′ velocity was similar in the 2 groups (*p*: 0.078). The C-Tor group had higher total cholesterol and LDL (*p*: 0.003 and 0.006 respectively). All C-Tor patients undergoing stress tests had positive results. The only independent C-Tor predictors in a regression analysis were HTN, total cholesterol, A-wave velocity, and deceleration time (DT) (odds ratio: 14.7, 1.03, 1.15, and 0.95, all *p*: < 0.05). A-wave velocity had the best area under the curve, sensitivity, and specificity for C-Tor prediction (0.88, 73.3%, and 96.7% respectively) followed by DT (0.86, 66.67%, and 96.6% respectively).

**Conclusion:**

C-Tor is associated with increased C-IMT, HTN, hyperlipidemia, and left ventricular diastolic dysfunction; all contributing to an ongoing atherosclerotic process. A-wave velocity and DT were independent predictors of C-Tor. C-Tor may cause microvascular ischemia that merits further investigation.

## Background

Coronary artery disease (CAD) is a major cause of mortality and morbidity worldwide [1], and the gold standard for its diagnosis is coronary angiography (CAG). Coronary tortuosity (C-Tor) is a common finding during CAG but there are conflicting data about its link to atherosclerosis: one study [2] found a negative correlation with CAD, although previous studies linked it to age and hypertension (HTN)—important CAD risk factors [3–5]. The resultant shear forces may enhance the growth and the ensuing rupture of atheromatous plaques, resulting in acute coronary syndromes [6, 7].

Carotid intima-media thickness (C-IMT) has been used as a surrogate for CAD. It signifies early atherosclerosis and smooth muscle proliferation. There is also a graded increase in cardiovascular risk with increasing C-IMT [8]. A C-IMT ≥ 0.9 mm is abnormal, but this differs with age [9–12].

Impaired left ventricular relaxation—as a function of diastolic dysfunction—is also a frequent finding in echocardiography studies and is associated with advanced age [13], obesity [14, 15], HTN [16, 17], diabetes mellitus (DM) [18, 19], and dyslipidemia [20]: the main risk factors of CAD. Its presence is independent of the presence of CAD. However, its relationship with C-Tor has limited data.

In this study, we investigated the interplay between C-Tor, C-IMT, diastolic dysfunction, and the other traditional risk factors of atherosclerosis (HTN, DM, dyslipidemia, and smoking) in patients with non-significant CAD.

## Methods

### Study design

This was a retrospective case-control study that included patients who underwent CAG in a tertiary-care hospital between July 2017 and June 2018. Table 1 lists the exclusion criteria. Patients with C-Tor were compared with an age and gender-matched control group with normal coronaries. All patients eligible for participation signed an informed consent.

**Table 1:** Exclusion criteria of the study cohort

Patients were excluded if they had any of the following:	
Significant CAD (≥ 50% diameter stenosis) in coronary angiography	
Acute coronary syndromes	
History of coronary artery revascularization (CABG or PCI)	
Dilated cardiomyopathy	
Permanent pacemakers	
Persistent arrhythmias	
Significant valvular disease	
Uncontrolled hypertension	
Chronic kidney disease	
Severe chronic lung disease	
Severe anemia	

*CAD* coronary artery disease, *CABG* coronary artery bypass grafting, *PCI* percutaneous coronary intervention

### Clinical risk factors

In all subjects, a fasting blood sample was collected before the CAG for lipid and blood sugar analysis. We defined the clinical risk factors as follows: dyslipidemia: total plasma cholesterol level ≥ 200 mg/dL or a low-density lipoprotein (LDL) cholesterol ≥ 130 mg/dL or on lipid-lowering drugs at the time of the investigation; HTN: systolic blood pressure ≥ 140 mmHg, or diastolic blood pressure ≥ 90 mmHg; or taking antihypertensive medications; DM: fasting plasma glucose ≥ 126 mg/dl, or on anti-diabetic agents. The smoking status was noted: current or ex-smokers versus non-smokers. Obesity was defined as a body mass index ≥ 30 kg/m^2^.

### Coronary angiography

**Fig. 1:**
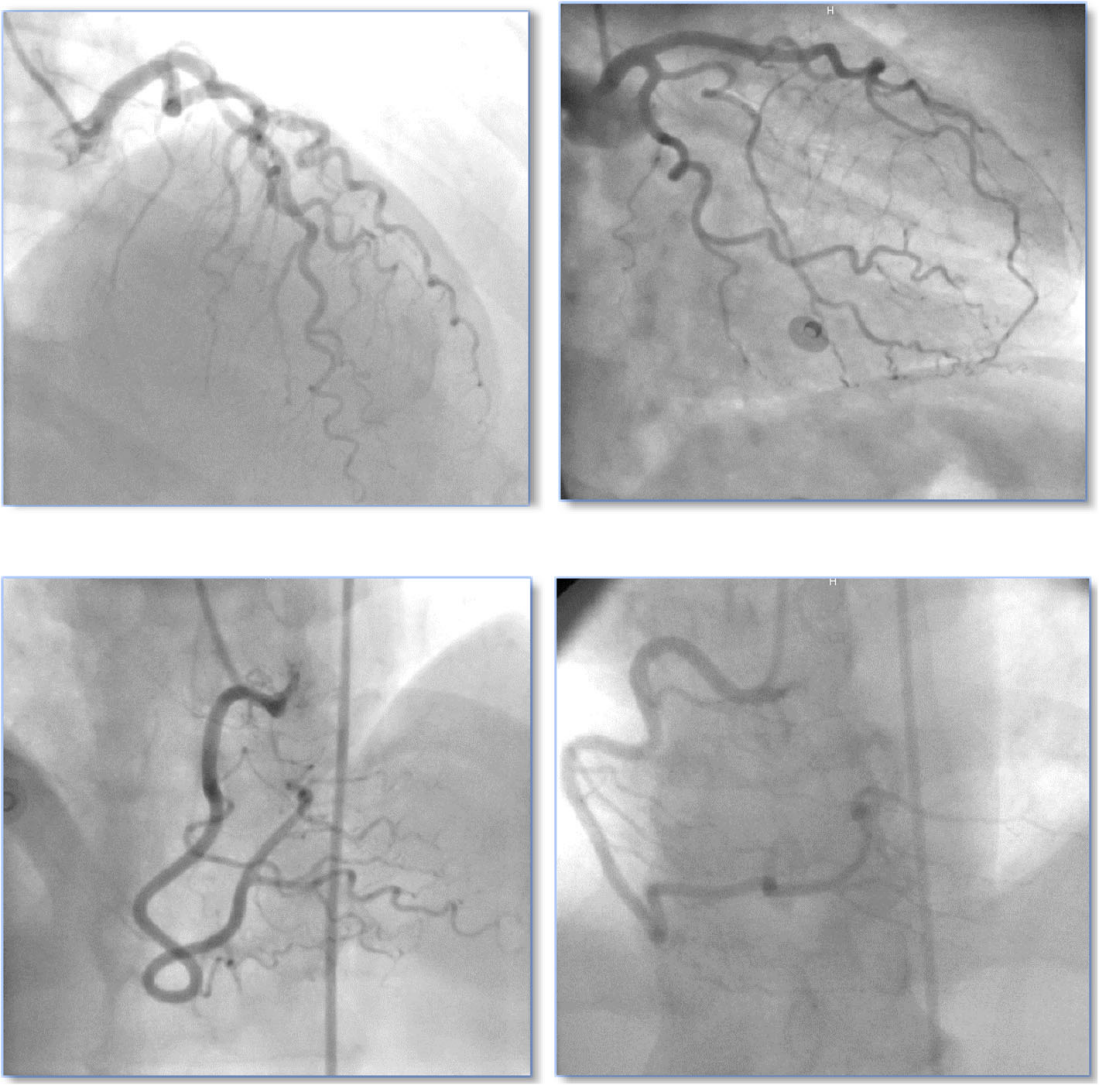
Coronary angiograms for different patients showing tortuosity in anteroposterior view with cranial angulation

Selective diagnostic CAG was performed with Judkin’s technique in right/left anterior oblique and anteroposterior views with different cranial and caudal angulations. The main coronary arteries (left anterior descending artery, left circumflex artery, and right coronary artery) were tracked. C-Tor was defined as the presence of ≥ 3 bends (≥ 45° change in the vessel direction along its main trunk) in at least one of the main coronary arteries in CAG [21] (Fig. 1).

### Carotid intima-media thickness

**Fig. 2:**
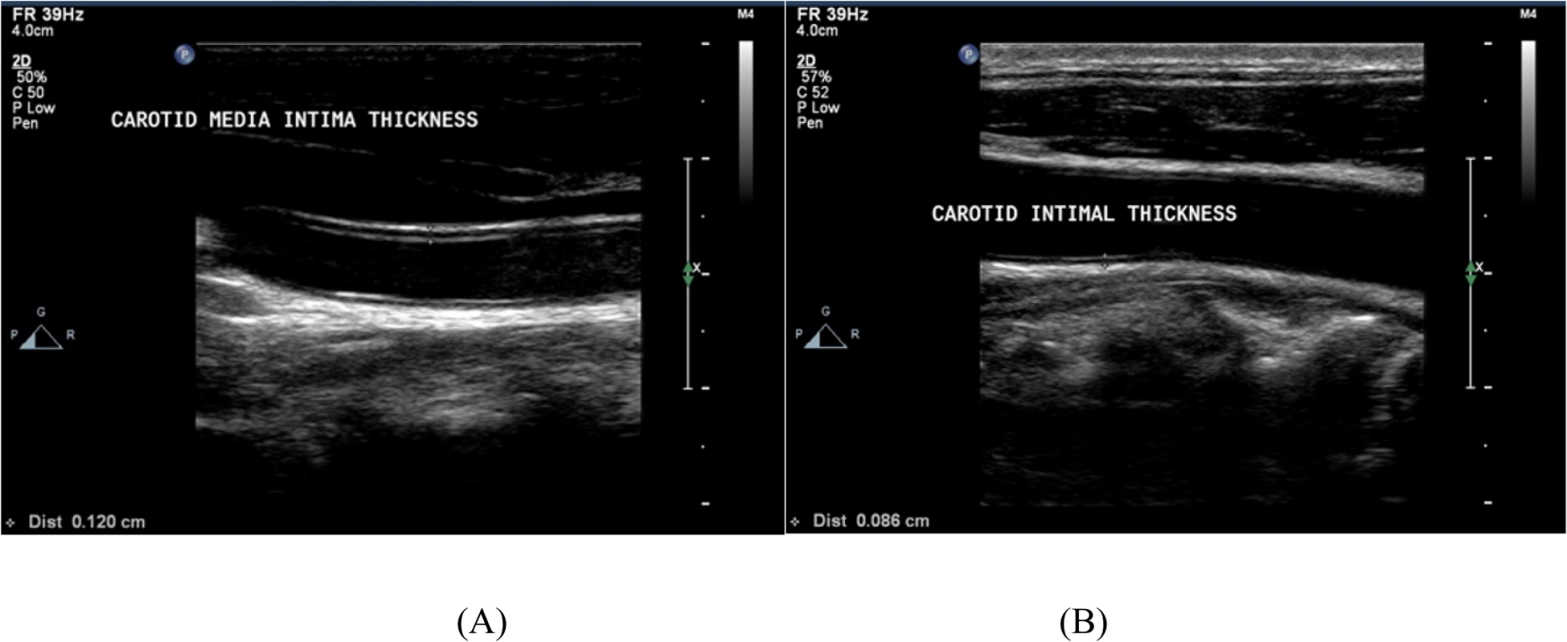
C-IMT measurement. Patient (**a**) had a normal C-IMT (0.12 mm). Patient (**b**) had an increased C-IMT (0.86 mm). C-IMT: Carotid intima-media thickness

We used the Philips IE 33 ultrasound system (Philips Medical Systems, Andover, MA, USA) and a 5–10 MHz multi-frequency high-resolution linear transducer to measure the maximum C-IMT from the common carotid artery, with the patient on his back, his neck extended, and his head slightly turned to the opposite side of the carotid artery being examined. A 10-mm longitudinal section was studied at a distance of 1 cm from the carotid bifurcation. The intima-media and the media-adventitia interfaces were measured in the proximal and distal walls in the anterior, lateral, and posterior projections, along an axis perpendicular to the artery. Six measurements were obtained in each carotid artery, to record the maximum. We considered C-IMT ≥ 0.9 mm abnormal [22] (Fig. 2).

### Transthoracic echocardiography

**Fig. 3:**
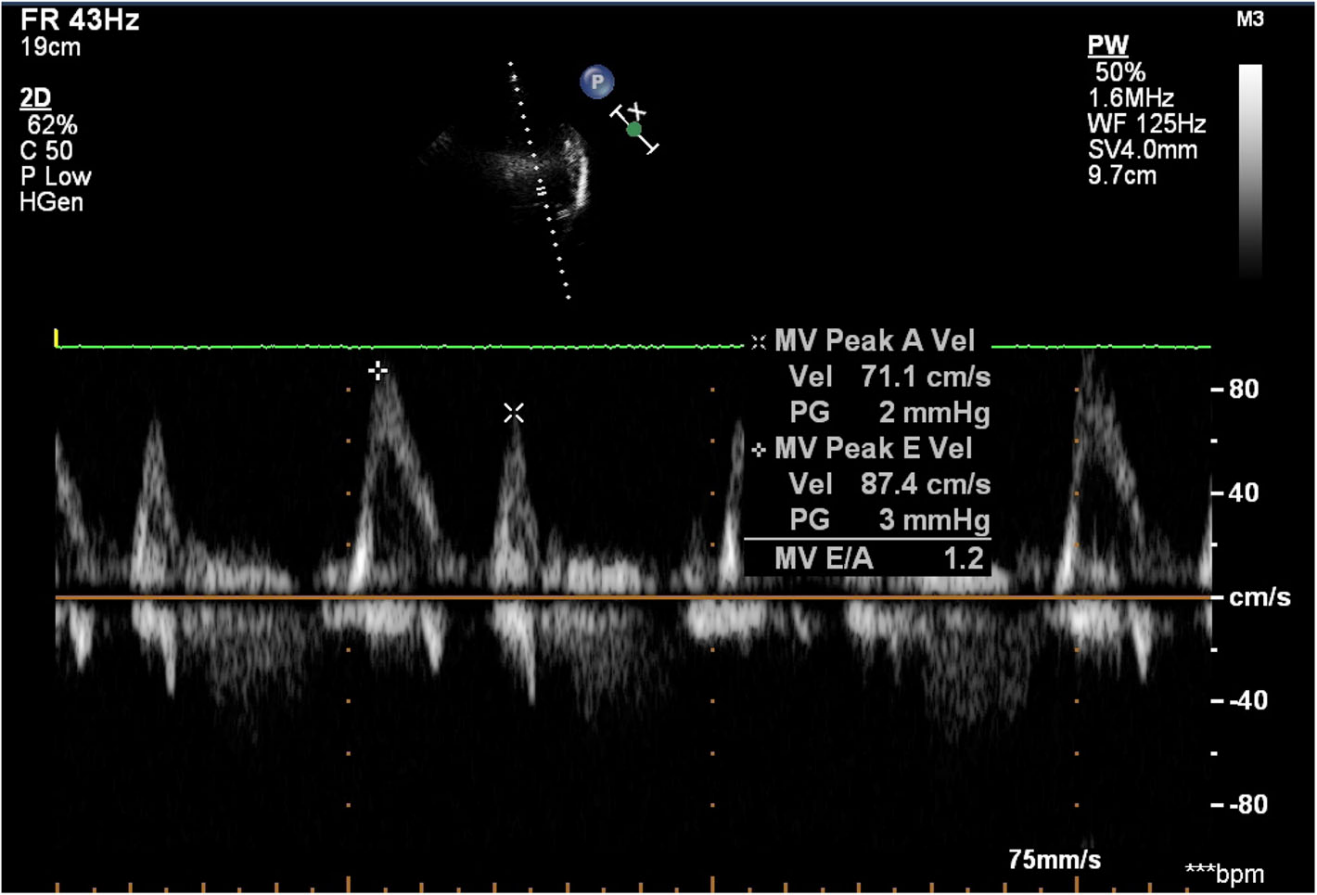
Assessment of diastolic function by transmitral inflow pulsed wave Doppler study

**Fig. 4:**
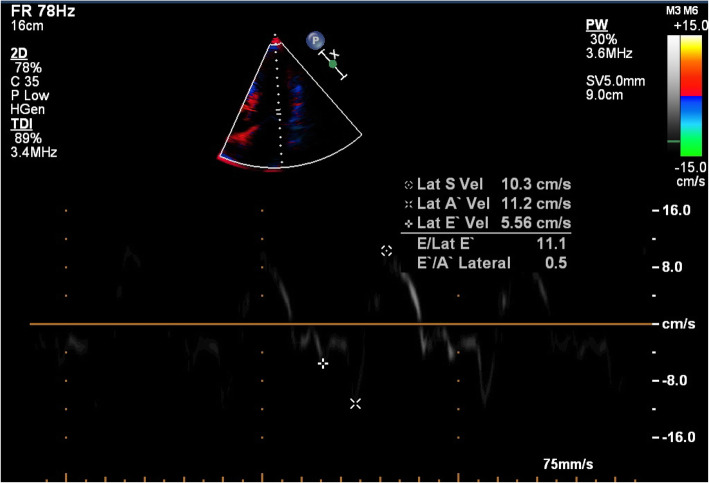
Assessment of diastolic function by lateral mitral annulus tissue Doppler imaging

A standard transthoracic echocardiography study was done on all patients using the same Philips IE 33 ultrasound system, in the left lateral recumbent position during quiet breathing. Pulsed-wave Doppler evaluation of the mitral inflow was acquired in the apical 4 chamber view, with the sample marker at the tips of the mitral valve leaflets. Doppler measurements were averaged over three consecutive cardiac cycles: peak early (E) and late (A) transmitral inflow velocities (in cm/s), the ratio of early to late peak velocities (E/A), and the deceleration time (DT) in ms [23] (Fig. 3). The grades of diastolic dysfunction were grade I (impaired relaxation pattern; defined as E/A < 1 and DT > 220 ms), grade II (pseudo-normalized pattern; E/A > 1 with DT between 160 and 220 ms, with ≥ 0.5 or reversal of the ratio with Valsalva maneuver), grade III (restrictive pattern; E/A > 1 and DT < 160 ms) [23]. We considered patients with normal diastolic function and grade I diastolic dysfunction as insignificant, while patients with grade II and III as having significant diastolic dysfunction. We also performed tissue Doppler imaging (TDI) from a sample volume positioned at (or 1 cm within) the lateral mitral annulus in the apical 4 chamber view and adjusted—if needed—to cover the longitudinal motion of the annulus in systole and diastole. The wall filter was set at 100 Hz to exclude high-frequency signals and the Nyquist limit to a velocity scale of 20 cm/s above and below the zero-velocity baseline. Gains were minimized to get a clear tissue signal with the least background noise. Annular motions were recorded at a frame rate of 80 to 140 frames per second and a sweep speed of 75 to 100 mm/s to achieve the best spectral display of myocardial velocities. The parameters measured were the early (E′) diastolic velocity, the late (A′) diastolic velocity, E′/A′, E/E′, and peak systolic velocity (S) [24] (Fig. 4).

### Statistical analysis

Data analysis was performed using the SPSS 23.0 statistical package for Windows (SPSS Inc., Chicago). All continuous variables were normally distributed, so were presented as mean ± standard deviation (SD). Categorical data were presented as numbers (percentages). Chi-square test was used to compare categorical variables while independent *t* tests were used for comparing continuous variables. Significant variables in the univariate analysis entered a multivariate stepwise logistic regression analysis to detect the independent predictors of C-Tor (outcome).

Then, receiver operating characteristics curve (ROC) analysis was done for the independent variables to derive the cutoff value with maximum sensitivity and specificity. The area under the ROC curve (AUC) was reported as an index of accuracy. In all cases, statistical significance is accepted at a *p* value < 0.05.

## Results

**Fig. 5:**
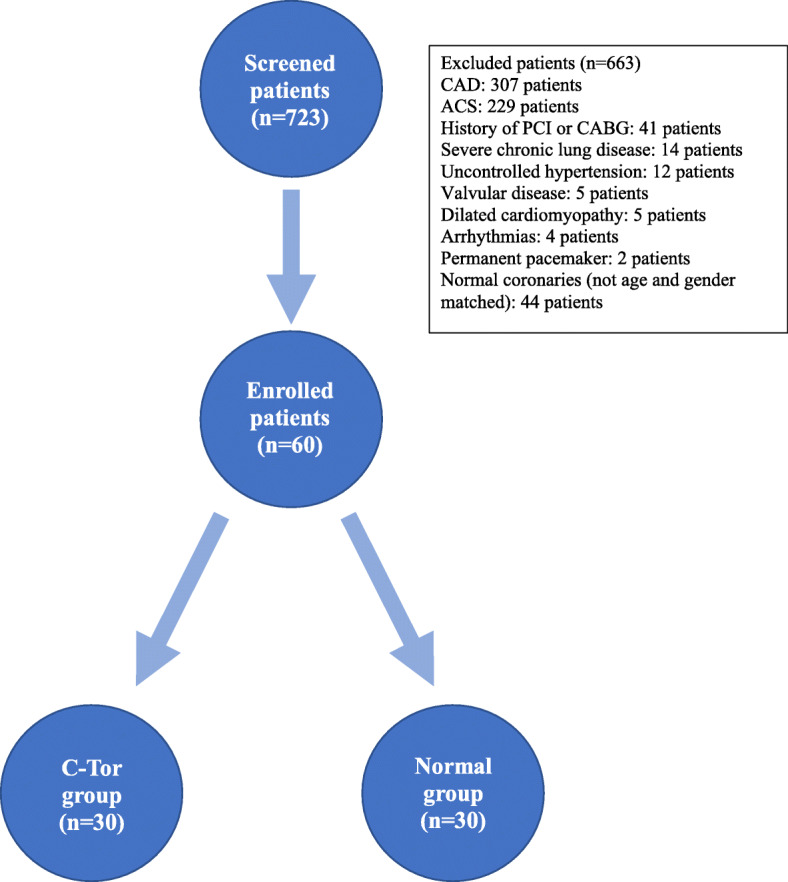
Flow chart of the patients in the study. *CAD* coronary artery disease, *PCI* percutaneous coronary interventions, *CAG* coronary artery bypass grafting, *C*-*Tor* coronary tortuosity

We screened 723 patients undergoing CAG: 663 were excluded based on the exclusion criteria, and 30 patients between July 2015 and June 2016 fulfilling the criteria of C-Tor and were compared with an age and gender-matched control group with normal coronaries during the same period (Fig. 5).

The baseline clinical and demographic characteristics were similar in the two study groups (Table 2) except HTN, which was significantly more common in the C-Tor group (86.7% vs. 30%, *p* < 0.002).

**Table 2:** Baseline demographics and clinical characteristics of the patients with and without coronary tortuosity

Characteristic	C-Tor (*n* = 30)	Normal (*n* = 30)	*P* value
Age (years)	57 ± 7.9	57 ± 7.9	1.0
Male gender	9 (30%)	9 (30%)	1.0
HTN	26 (86.7%)	9 (30%)	**< 0**.**001**
DM	10 (33.3%)	10 (33.3%)	1.0
Smokers	6 (20%)	8 (26.7%)	0.54
Typical chest pain	14 (46.7%)	16 (53.3%)	**< 0**.**001**
Positive stress testª	13 (100%)^b^	1(100%)^b^	**NA**

*C-Tor* coronary tortuosity, *HTN* hypertension, *DM* diabetes mellitus, *NA* not applicable

ªStress tests included stress electrocardiograms, stress myocardial perfusion imaging, or stress echocardiography

^b^All patients who did a stress test were positive, the others did not do the test (no negative test results)

The C-IMT was significantly higher in the C-Tor group, reaching the cutoff of 9 mm which we considered abnormal. The individual parameters of diastolic dysfunction were significantly different between the two groups: the E velocity and DT were significantly lower in the C-Tor group, while the E/E′ ratio, A, and A′ velocities were significantly higher. The E/A ratio was < 1 in the C-Tor group and > 1 in the normal group. The S′ velocity—which is not a parameter of diastolic function—was similar between the 2 groups (Table 3).

**Table 3:** Echocardiographic and Doppler parameters of the patients with and without coronary tortuosity

Parameter	C-Tor (*n* = 30)	Normal (*n* = 30)	*P* value
C-IMT (mm)	9 ± 7	8 ± 9	**0.007**
E velocity (cm/s)	75.6 ± 13.9	87.6 ± 9.7	**0.001**
A velocity (cm/s)	83 ± 20.24	57.5 ± 8.4	**< 0**.**001**
E/A ratio	0.99 ± 0.39	1.54 ± 0.23	**< 0**.**001**
DT (ms)	137 ± 43.7	184 ± 16.25	**< 0**.**001**
S′ velocity (cm/s)	9.15 ± 1.36	8.81 ± 1.03	0.078
E′ velocity (cm/s)	9.8 ± 3.07	12.6 ± 2.55	**< 0**.**001**
A′ velocity (cm/s)	10.6 ± 2.2	8.35 ± 2.2	**< 0**.**001**
E/E′ ratio	8.1 ± 1.59	6.9 ± 1.3	**0.005**
E′/A′ ratio	1.01 ± 0.54	1.66 ± 0.31	**< 0**.**001**
Diastolic dysfunction^a^	21 (70 %)	0 (0 %)	**< 0**.**001**

*C*-*Tor* coronary tortuosity, *C*-*IMT* carotid intima-media thickness, *DT* deceleration time

^a^Diastolic dysfunction was considered significant for grades II and III

The C-Tor group had higher total cholesterol and LDL, and there was also a trend for higher HDL and triglycerides (Table 4).

**Table 4:** Lipid profile parameters of the patients with and without coronary tortuosity

Parameter	C-Tor (*n* = 30)	Normal (*n* = 30)	*P* value
Total cholesterol (mg/dL)	193 ± 39.5	163 ± 25.8	**0**.**003**
LDL Cholesterol (mg/dL)	112 ± 34.3	93 ± 16.7	**0**.**006**
HDL Cholesterol (mg/dL)	40 ± 7.5	38 ± 4.3	0.05
Triglycerides (mg/dL)	175 ± 121.5	138 ± 36.7	0.06

*C-Tor* coronary tortuosity, *LDL* low-density lipoproteins, *HDL* high-density lipoproteins

**Fig. 6:**
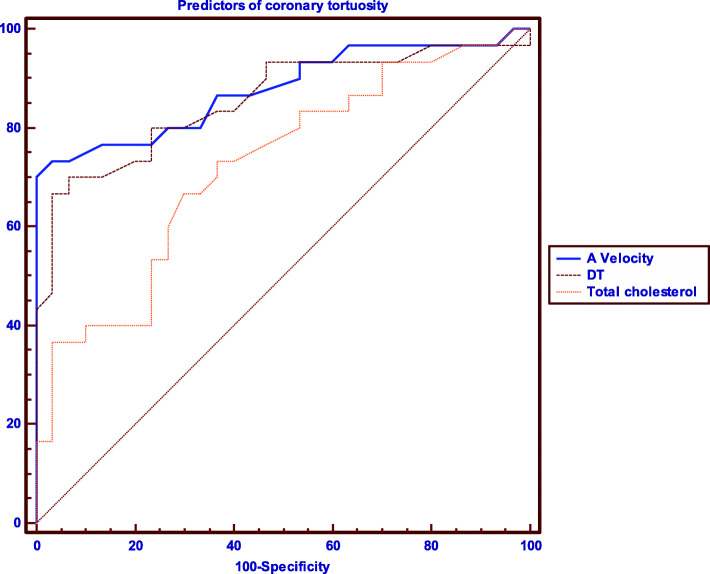
ROC analysis of the most significant predictors of coronary tortuosity. A wave velocity had the highest area under the curve, followed by DT and total cholesterol. *DT* deceleration time

A multivariate stepwise regression analysis of the significant variables showed that the only independent predictors of C-Tor were HTN, total cholesterol, A wave velocity, and the DT (Table 5). A wave velocity had the best AUC in the ROC analysis and the highest sensitivity and specificity for C-Tor prediction, followed by DT (Table 6, Fig. 6). E/A ratio and diastolic dysfunction were removed from the analysis because of high collinearity.

**Table 5:** Multivariate stepwise regression analysis for clinical and echocardiographic predictors of coronary tortuosity

Variable	OR	95% CI	*P* value
Clinical			
HTN	14.7	3.5-62.1	**< 0**.**001**
Total Cholesterol	1.03	1.01-1.05	**0**.**018**
Echocardiographic			
A velocity (cm/s)	1.15	1.05-1.25	**0**.**002**
DT (ms)	0.95	0.92-0.99	**0**.**005**

*OR* odds ratio, *CI* confidence interval, *HTN* hypertension, *DT* deceleration time

**Table 6:** ROC analysis for clinical and echocardiographic predictors of coronary tortuosity

Variable	AUC (95% CI)	*P* value	Sensitivity (95% CI)	Specificity (95% CI)
Clinical				
Total Cholesterol (> 168 mg/dL)	0.72 (0.59–0.83)	< 0.001	73.3% (54.1–87.7)	63.3% (43.9–80.0)
Echocardiographic				
A velocity (> 72.8 cm/s)	0.88 (0.77–0.95)	< 0.001	73.3% (54.1–87.7)	96.7% (82.7–99.4)
DT (≤ 155 ms)	0.86 (0.74–0.93)	< 0.001	66.67% (47.2–82.7)	96.6% (8.7–99.4)

*ROC* receiver-operator characteristics, *AUC* area under the curve, *CI* confidence interval, *DT* deceleration time

## Discussion

The key result of our research is that C-IMT, HTN, dyslipidemia, and left ventricular diastolic dysfunction were more prominent in patients with C-Tor than in those without C-Tor.

To our knowledge, this is the first study to investigate the relationship between C-IMT and C-Tor. C-IMT is a surrogate of CAD [25, 26], whereas C-Tor induces local low shear stresses that can influence atherosclerosis formation and plaque stability through altering endothelial cell function and gene expression [27, 28]. In our study, even after excluding patients with significant CAD, C-IMT was still higher in the C-Tor group—the atherosclerotic process is in action, although not yet causing significant CAD.

C-Tor has also been linked to the changes in the left ventricular geometry—namely concentric hypertrophy [29, 30]. This explains its association with diastolic dysfunction. Hypertrophy may induce C-Tor through blood flow changes, wall stress, and growth factors [31, 32]. HTN is commonly associated with diastolic dysfunction [16, 33] and can cause C-Tor through the induced hypertrophy. A previous study [21] found a similar association with impaired relaxation pattern on echocardiography, but not with HTN. The E/A ratio and IVRT were significant independent predictors of C-Tor on multivariate analysis—as opposed to the A wave velocity and DT in our study. They enrolled more patients (*n* = 104), but their study design was not age/gender-matched with a control group. They also did not perform a TDI study—although this proved to be not a significant predictor of C-Tor in our study.

The association between C-Tor and atherosclerosis has conflicting data. A more recent study [2] on 367 non-CAD patients found that all the CAD risk factors, including HTN and dyslipidemia, were not associated with C-Tor, and that female gender and current non-smoking status were the only significant determinants for C-Tor. However, HTN was a significant predictor of C-Tor in the whole patient cohort (CAD and non-CAD patients). Age and female gender were associated with C-Tor in a previous study [21] but in our study, we nullified the effect of these factors by an age and gender-matched case-control design. The same study [2] also found a negative correlation between C-Tor and CAD, although previous studies linked it to age and hypertension (HTN): which are important CAD risk factors [3, 5, 34–36]. The resultant shear forces in the tortuous vessels may enhance the growth and the ensuing rupture of atheromatous plaques, resulting in acute coronary syndromes [7, 37, 38]. This was proven in an earlier study on the femoral artery [35]. However, a more recent study disproved this concept in the coronary vessels [39].

This study was done on elective patients; some of them underwent stress testing before the CAG. All patients with C-Tor who had an earlier stress test showed a positive test result for ischemia while having insignificant CAD. This may denote ischemia at a microvascular level, which suggests that C-Tor is not entirely benign.

The mechanism of C-Tor is still not fully understood. It may occur due to reduced axial strain which results in abnormal MMP activity [40]. It can also be an adaptation to high flow and high shear stress [41]. This deserves further investigation.

### Study limitations

The small sample size of the study cohort is one limitation: the patients were enrolled consecutively within a pre-specified timeframe, but the stringent exclusion criteria, especially the CAD criterion excluded many of them. Invasive imaging of the coronary arteries (e.g., intravascular ultrasound) would have given a better insight into the coronary vascular structure. Finally, the assessment of the local shear stress created by the C-Tor with the use of a flow dynamic model would be of great value in a better understanding of the underlying pathophysiology.

## Conclusion

C-Tor is associated with increased C-IMT, HTN, hyperlipidemia, and left ventricular diastolic dysfunction—all contributing to an ongoing atherosclerotic process. A wave velocity and DT were the most significant independent predictors of C-Tor. C-Tor may cause microvascular ischemia, which deserves further investigation.

## Data Availability

The datasets used and/or analyzed during the current study are available from the corresponding author on reasonable request.

## Abbreviations

CAD: Coronary artery disease
CAG: Coronary angiography
C-Tor: Coronary tortuosity
HTN: Hypertension
C-IMT: Carotid intima-media thickness
DM: Diabetes mellitus
CABG: Coronary artery bypass grafting
PCI: Percutaneous coronary intervention
LDL: Low-density lipoprotein
DT: Deceleration time
TDI: Tissue Doppler imaging
NA: Not applicable
HDL: High-density lipoprotein
OR: Odds ratio
CI: Confidence interval
ROC: Receiver-operator characteristics
AUC: Area under the curve
